# Bird Biology and Conservation

**DOI:** 10.3390/biology15161358

**Published:** 2026-08-10

**Authors:** Alessandro Ferrarini

**Affiliations:** Lipu-BirdLife Italy, Via Udine 3/a, I-43122 Parma, Italy; sgtpm@libero.it; Tel.: +39-0521-1910728

## 1. Introduction

There is an increasing need to develop and test new theoretical frameworks, methodologies, and field survey approaches for promoting more satisfactory conservation of bird species. Accordingly, this Special Issue hosts both original research and synthetic reviews with regard to different biological levels (population, species, community, metacommunity), including, but not limited to, the following: (1) bird field sampling methods; (2) impacts of climate change; (3) multiscale nest-site selection; (4) reliable estimates of population sizes and their temporal trends; (5) methods for stopover site identification of migration birds; (6) simulation-based conservation strategies; (7) habitat restoration; (8) methods for estimating the ecological performance of migratory bird conservation.

## 2. Special Issue Overview

Reyes et al. [1] studied the social networks of three populations of the endangered Floreana Mockingbird, and used model simulations of different translocation scenarios to test the effects of social disruption on such social networks. Simulated removals demonstrated that the loss of individuals occupying different network positions produced variable levels of social disruption. This article emphasizes the importance of dominance hierarchies and group structure on the effects of selectively removing individuals from populations of endangered species.

Zhao et al. [2] analyzed the combined effects of climate change and inter-specific competition on the spatial distribution of Elliot’s Pheasant in Huzhou, China. Their findings highlight both the high climate sensitivity and competitive vulnerability of this species. From a methodological viewpoint, I emphasize their bird field sampling method, based on 97,000 camera-days of infrared monitoring from 2019 to 2022.

Aymá et al. [3] analyzed nest-site selection of Burrowing Owl across multiple spatial scales. Their results suggest that nest-site selection emerges from interactions among spatial scales rather than from isolated factors, which supports findings from the hierarchical habitat-selection theory. From a methodological viewpoint, I draw attention to their integrated multiscale model which showed substantially greater support and discriminatory power than single-scale models.

Zhao et al. [4] analyzed habitat utilization of the Temminck’s Tragopan. Habitat utilization is a critical determinant of animal survival and reproductive success. Their analysis revealed a significant preference for sites characterized by greater tree and bamboo height, higher canopy and bamboo cover, increased litter coverage, and taller shrub layers. In contrast, the species consistently avoided locations dominated by dense, tall herbaceous vegetation.

Tella et al. [5] compared three methods for estimating abundances of the globally endangered African Grey Parrot. They conducted a nationwide survey of this species in Equatorial Guinea using walk transects, car transects, and point counts, which yielded 1166 encounters and 2972 recorded individuals. Their findings support the use of the three methods, either individually or in combination, to estimate the abundance of this globally endangered species.

Fang et al. [6] propose an enhanced bird detection model named Birds-YOLO to address the challenges posed by complex background interference, varying target sizes, and high species diversity in bird detection tasks. Their model is validated through extensive ablation studies and comparative experiments, indicating its strong potential for practical deployment in bird detection tasks in complex natural environments.

Zulfiqar et al. [7] assessed human-perceived threats to cranes (*Grus virgo* and *Grus grus*) along Pakistan’s Indus Flyway using 400 stakeholder questionnaires. Their results align with global evidence of uniform crane threats demanding region-specific enforcement, community-led anti-poaching initiatives, and targeted awareness programs to shift high-threat communities toward crane-friendly coexistence practices.

Tozetti et al. [8] describe the corneal histomorphometry of eight birds from different species. Their findings contribute to a better understanding of the corneal morphology in diverse bird species, and may support future ophthalmic or comparative anatomical research.

Stanek et al. [9] analyzed the impacts of drought and stand-replacing wildfires on the composition of avian communities. Their results suggest that, although bird communities are immediately impacted by fire-driven changes, they can rebound over time, but it is unclear how long-term drought may continue to shape the composition of these avian communities.

The study by Kopij [10] analyses the interrelated issues related to population density, territoriality, and philopatry in shrikes inhabiting an acacia savanna in northern Namibia. Results show marked interspecific differences in territoriality and philopatry. Furthermore, even within the same species, marked temporal differences occurred in population density.

Marnn et al. [11] evaluated the effects of restoration on the populations of waterbirds in Myanmar’s Moe Yun Gyi wetland by analyzing the composition and the spatiotemporal dynamics of waterbirds communities, in particular changes in diversity. They find that community’s richness has partially recovered, but seasonal water level volatility continues to impact diversity, and the application of restoration techniques directly altered the extent and depth of water bodies. They propose implementing a rational system for managing water levels, optimizing wetland hydrology, and enhancing water level regulation to safeguard significant resting areas along migration paths.

Da Silva and Matsushita [12] show how Japanese tits (*Parus minor*) use wing-fluttering movements to transmit an “after you” directive to their partners, implying a degree of cognitive skill previously thought to be unique to humans and great apes. They suggest that wing fluttering functions are an effective communicative gesture in this species, influencing male nesting behavior.

He et al. [13] propose the T-DBSCAN algorithm, an enhanced clustering model, for stopover site identification of migration birds based on satellite positioning data. Results demonstrate that the algorithm outperforms conventional habitat identification accuracy, and can also manage large amounts of discontinuous satellite tracking data.

Ferrarini et al. [14] analysed bird richness and abundance for an Italian Town, and proposed simulation-based conservation strategies. Results show that local planners can largely influence bird richness in the study area, with both positive (urban greening) and negative (urban densification and sprawl) strategies. From a methodological viewpoint, I emphasize their methodological approach to test the degree of sampling completeness and environmental filtering.

Song et al. [15] quantified the ecological performance of migratory bird conservation in the Poyang Lake Wetlands in China. Their findings indicate that ecological connectivity is essential for the conservation of migratory bird habitats, and the establishment of ecological corridors can help reconcile conflicts between conservation efforts and development objectives.

The review by Wang et al. [16] analysed secondary contact zones, i.e., areas where incipient species or divergent populations may meet, mate, and hybridize. This review exhaustively summarizes the variations in unique traits (vocalization, plumage, beak, and migratory traits) and the various movement patterns of secondary contact zones observed in previous publications.

The case report by Komarowska et al. [17] sets out to examine the status of sympatric populations of south polar and brown skuas at two sites on King George Island, Antarctica. The study sites were designated as Important Bird and Biodiversity Areas (IBAs). The expansion of both IBA boundaries is recommended based on this study.
